# Hypofractionated Stereotactic Radiotherapy after Transarterial Chemoembolisation Failure in an Unresectable Hepatocellular Carcinoma: A Case Presentation

**DOI:** 10.1155/2013/146215

**Published:** 2013-03-10

**Authors:** Francesco Fiorica, Carlo Greco, Sergio Boccia, Sergio Sartori, Antonio Stefanelli, Francesco Cartei, Stefano Ursino

**Affiliations:** ^1^Department of Radiation Oncology, University Hospital “S. Anna,” Corso Giovecca 203, 44100 Ferrara, Italy; ^2^Department of Radiation Oncology, University Hospital “S. Chiara,” Via Roma 67, 56126 Pisa, Italy; ^3^Department of Gastroenterology, University Hospital “S. Anna,” Via Aldo Moro 8, 44124 Ferrara, Italy; ^4^Department of Internal Medicine, Section of Interventional Ultrasound, University Hospital “S. Anna,” Via Aldo Moro 8, 44124 Ferrara, Italy

## Abstract

*Introduction*. Transarterial chemoembolization is the first-line treatment in unresectable hepatocellular carcinoma. There is no standard treatment after transarterial chemoembolization failure. We report the case of a patient with advanced hepatocellular carcinoma who showed a complete response and a long cancer control with hypofractionated stereotactic radiotherapy after transarterial chemoembolization failure. *Case Presentation*. A 70-year-old Caucasian woman was treated with transarterial chemoembolization for advanced hepatocellular, but no cancer control was obtained. A hypofractionated stereotactic radiotherapy was planned delivering 40 Gy in 5 fractions. A dramatic reduction in alpha-fetoprotein was observed. Contrast-enhanced ultrasonography at 1 and 2 months showed large necrotic areas. Computerised tomography scan showed a 90% objective tumour response, then a complete remission at 3 and 6 months after treatment, respectively. Status of patient remained unchanged for 2 years. *Conclusions*. Hypofractionated stereotactic radiotherapy can improve survival and prognosis of unresectable hepatocellular carcinoma patient.

## 1. Introduction

Hepatocellular carcinoma (HCC) is a very heterogeneous disease, and the management of therapeutic approach may be variable and strongly related to patient's liver dysfunction as well as tumour stage [1]. A patient affected by intermediate or advanced stage HCC is only candidate for a palliative treatment. Transarterial chemoembolization (TACE) is standard treatment of unresectable HCC [2]; however, prognosis of these patients remains dismal, especially when TACE fails. Hypofractionated stereotactic radiotherapy (HSRT) can lead to a significant benefit for patients affected by intermediate or advanced HCC, alone or combined with TACE [3].

We report the case of a patient with advanced HCC who showed a complete response and a long cancer control with HSRT after TACE failure.

## 2. Case Presentation

A 70-year-old woman, with a history of alcoholic cirrhosis was admitted to our hospital because a liver mass had been discovered by ultrasonography scan. Her Eastern Cooperative Oncology Group (ECOG) performance score was 1. Physical examination showed no palpable mass, abdominal distension, or weight loss. Laboratory details showed white blood cell 5,980/*μ*L; red blood cell 4,24 × 10^6^/*μ*L; haemoglobin 10,8 g/dL; hematocrit 35%; platelet 137,000/*μ*L; INR 1,11; urea nitrogen 26 mg/dL; creatinine 0,73 mg/dL; total protein 7,8 g/dL; albumin 3,5 g/dL; aspartate aminotransferase (AST) 55 U/L; alanine aminotransferase (ALT) 42 U/L; alkaline phosphatase (ALP) 500 U/L; gamma-glutamyl transpeptidase (GGT) 264 U/L; cholinesterase 5238 U/L; total bilirubin 0,64 mg/dL; alpha-fetoprotein (-FP) 319 ng/mL. Gastroscopy showed esophageal varices grade F1-F2. Her Child-Pugh score was A, and Meld score was 7. Both contrast-enhanced ultrasonography (CEUS) and CT scan revealed an 8 × 5 × 7 cm sized arterial enhancing and delayed washout mass extended to the VII and VIII hepatic segments associated with underlying cirrhosis and without portal invasion, ascites, and splenomegaly. Tumor stage was Barcelona Clinic Liver Cancer (1) stage C. 

**Figure 1:**
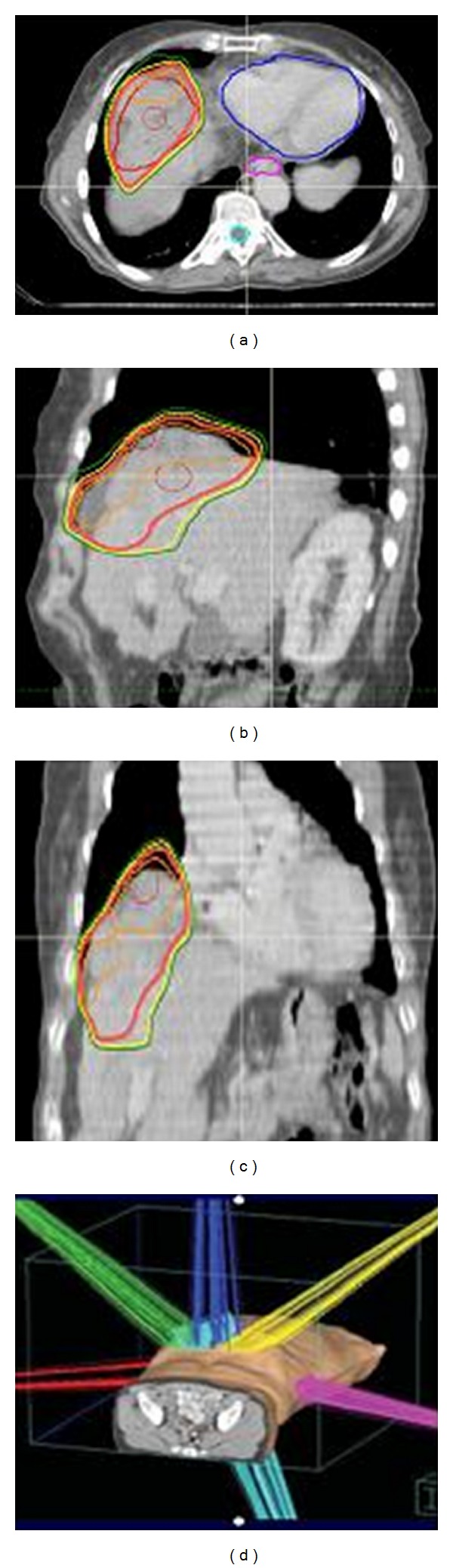
HSRT treatment plan: the axial (a), sagittal (b), coronal (c), and three-dimensional view (d). Red line: 100% isodose line; Yellow line: 95% isodose line; green line: 80% isodose line.

According to the practice guidelines of the American Association for the Study of Liver Disease (AASLD), transarterial chemoembolization (TACE) with epirubicin-lipiodol was performed. Thirty days after TACE, a noncontrast-enhanced CT showed no tumor shrinkage and a slight accumulation of lipiodol (pattern type IV). These data [4] evidenced an unsatisfactory response to TACE procedure. A hypofractionated stereotactic radiotherapy (HSRT) was performed. Patient was immobilized using the Elekta Stereotactic Body Frame which uses a rigid frame and a vacuum pillow and an abdominal compression device to reduce respiratory target motion. Treatment planning contrast-enhanced CT (3 mm slice thickness) was obtained to identify target volume visualization. According to our internal protocol based on the recent literature data (5), HSRT was delivered in 5 fractions for a total of 40 Gy on alternate days, and dose was prescribed to the 97% isodose line (Figure 1). As constrains for normal tissues, 33% and 50% of the whole liver volume received a total dose less than 21 Gy and 15 Gy, respectively; the percentage of each kidney volume to receive a total dose of 15 Gy was less than 35%, while the maximum total dose did not exceed 27 Gy for the spinal cord and stomach/small intestine and 30 Gy for heart. 

Planning was conducted on the Pinnacle 3D 8.0 m Version Planning System (Philips Healthcare, Best, Netherlands). The patient had no discomfort after the procedure, and no acute toxicity was reported. 

**Figure 2:**
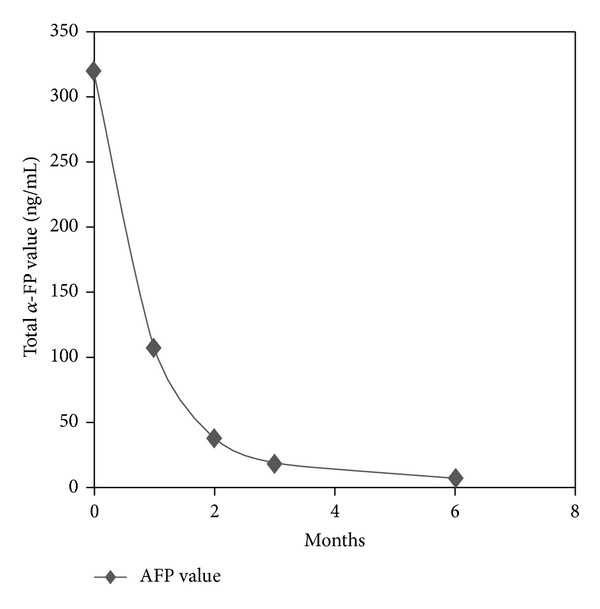
Changes in *α*-FP levels after HSRT.

**Figure 3:**
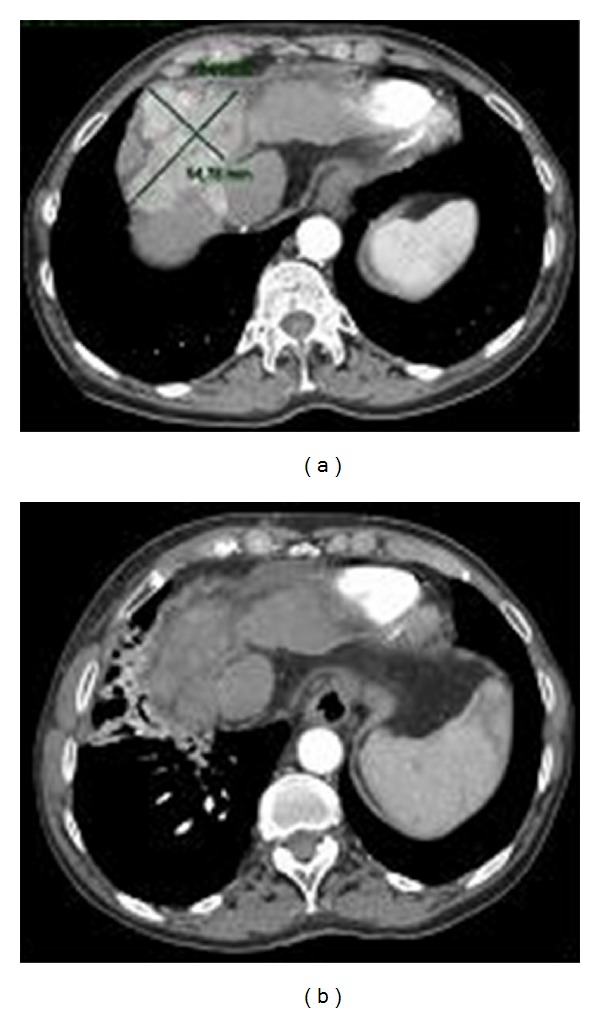
Abdominal computed tomography scans before and 6 months after HSRT show a complete disappearance of hypervascular arterial enhancement of primary tumour.

At 1, 2, and 3 months after HSRT, ALP, GGT, and a-FP decreased, respectively, to 411→ 400→361 U/L, 199→156→118 U/L, and 107,6→ 38→19,8 ng/mL; a further reduction of a-FP value at 7,4 ng/mL was observed within 6 months (Figure 2). CEUS at 1 and 2 months showed 3 large necrotic areas inside an unchanged size tumour mass. CT scan showed a 90% objective tumour response and a complete remission at 3 and 6 months after treatment, respectively (Figure 3). 

Twenty-four months following HSRT, patient showed a multifocal intrahepatic dissemination without evidence of “in-field” local recurrence; therefore a systemic therapy with sorafenib was planned.

## 3. Discussion 

According to AASLD, TACE is the first-line treatment in unresectable HCC. Nevertheless, it induces objective responses sustained for at least 6 months in only 35% of patients [5]. Although TACE can be repeated in most patients, therapeutic efficacy cannot be expected by repetitive procedure, so that there is no consensus regarding the optimum treatment/retreatment strategy [6]. Conversely, repeated TACE treatments can result in deterioration of the liver function [7]. Failure of TACE treatment can be predicted with a CT scan 1 month after TACE, by volume measurement and by retention of lipiodol within tumor tissue [8]. For patients who have failed locoregional therapy, systemic therapy is the standard of care. However, another locoregional treatment can be performed in order to improve locoregional tumour control probability.

The role of external beam radiotherapy has always been limited by the low-dose tolerance of liver to radiation and subsequent high risk of RILD due to the high percentage of liver irradiated [9].

However, latest technological developments in RT planning and treatment delivery, such as HSRT, have allowed us to deliver higher radiation tumour dose while sparing surrounding normal tissues, suggesting a significant benefit for patients affected by intermediate or advanced HCC, alone or combined with TACE, in order to improve clinical results.

Despite the high size of gross tumour mass, our patient experienced a long-term complete remission (2 years) and a subsequent “out-field” intrahepatic dissemination that required systemic therapy. Probably, the peripheral tumour localization and the good liver reserve have enabled us to safely deliver a tumoricidal dose without occurring major side effects.

Using radiotherapy, several monoinstitutional experiences have shown promising results with a high rate of complete (50–60%) and objective responses (75–80%) as well as low rate of severe toxicity [10–13]. Furthermore, when stereotactic radiotherapy is used, the pattern of hepatic “out-field” recurrence compared to local or distant failure seems to be the main cause of progression disease influencing patient life expectancy.

## 4. Conclusion

Our experience with this case demonstrates that a complete remission of primary and a good cancer control can be obtained in local advanced HCC patients with HSRT. Two year following HSRT, a multifocal intrahepatic dissemination was diagnosed without evidence of the primary HCC. This confirms that the pattern of hepatic “out-field” recurrence is the main cause of HCC progression. HSRT, after TACE failure, was very effective in our patient, suggesting that HSRT might improve the overall survival rate and provides a good prognosis for patients with unresectable HCC.
